# Alpha-synuclein oligomer-selective antibodies reduce intracellular accumulation and mitochondrial impairment in alpha-synuclein exposed astrocytes

**DOI:** 10.1186/s12974-017-1018-z

**Published:** 2017-12-11

**Authors:** Gabriel Gustafsson, Veronica Lindström, Jinar Rostami, Eva Nordström, Lars Lannfelt, Joakim Bergström, Martin Ingelsson, Anna Erlandsson

**Affiliations:** 1 Molecular Geriatrics, Department of Public Health and Caring Sciences, Rudbeck Laboratory, Uppsala University, SE-751 85 Uppsala, Sweden; 2 BioArctic AB, Warfvinges väg 35, 112 51 Stockholm, Sweden

**Keywords:** α-synuclein oligomers, Astrocytes, Antibodies, Mitochondria, Lysosomal degradation, Parkinson’s disease

## Abstract

**Background:**

Due to its neurotoxic properties, oligomeric alpha-synuclein (α-syn) has been suggested as an attractive target for passive immunization against Parkinson’s disease (PD). In mouse models of PD, antibody treatment has been shown to lower the levels of pathogenic α-syn species, including oligomers, although the mechanisms of action remain unknown. We have previously shown that astrocytes rapidly engulf α-syn oligomers that are intracellularly stored, rather than degraded, resulting in impaired mitochondria.

**Methods:**

The aim of the present study was to investigate if the accumulation of α-syn in astrocytes can be affected by α-syn oligomer-selective antibodies. Co-cultures of astrocytes, neurons, and oligodendrocytes were derived from embryonic mouse cortex and exposed to α-syn oligomers or oligomers pre-incubated with oligomer-selective antibodies.

**Results:**

In the presence of antibodies, the astrocytes displayed an increased clearance of the exogenously added α-syn, and consequently, the α-syn accumulation in the culture was markedly reduced. Moreover, the addition of antibodies rescued the astrocytes from the oligomer-induced mitochondrial impairment.

**Conclusions:**

Our results demonstrate that oligomer-selective antibodies can prevent α-syn accumulation and mitochondrial dysfunction in cultured astrocytes.

## Background

Cellular inclusions in the brain, referred to as Lewy bodies and Lewy neurites, are pathological hallmarks of Parkinson’s disease (PD) [1]. The inclusions predominantly consist of α-synuclein (α-syn) [2], a protein which aggregates into insoluble fibrils via the formation of soluble intermediates [3]. Such α-syn oligomers are particularly harmful [4] and have for example been shown to disrupt cellular membranes [5, 6] and induce mitochondrial dysfunction [7, 8].

Targeting pathological α-syn by either active or passive immunization has been shown to reduce α-syn levels and ameliorate behavioral symptoms in transgenic mouse models of synucleinopathy [9–13]. Due to their toxic nature, immunization against soluble α-syn oligomers is an especially attractive therapeutic target. Lewy body formation is believed to be protective rather than neurotoxic, and immunotherapy directed against fibrillar α-syn is therefore less likely to be effective. We have previously shown that treatment with antibodies directed against oligomeric α-syn lowers the CNS levels of oligomers in a transgenic α-syn-expressing mouse model [9]. However, the cellular mechanisms by which the antibodies act and which cell types are targeted remain unknown.

Although α-syn deposits are primarily found in neurons in the PD brain, they also appear frequently in astrocytes [14–19]. Being the most numerous glial cell type in the CNS, astrocytes have great impact on the brain environment and may constitute a very potent treatment target. Astrocytes play an important role in maintaining brain homeostasis [20], and their functions include metabolic support of neurons, modification of synapse signaling, recycling of neurotransmitters, blood brain barrier regulation, and glymphatic clearance [20–22]. In addition, astrocytes respond to neurodegenerative disorders, including PD, through astrogliosis, a process in which they convert to a reactive inflammatory state [23, 24]. Yet, the role of astrocytes in the development and progression of α-syn pathology has been infrequently studied. In a recent study, we investigated uptake, degradation, and toxic effects of soluble oligomeric α-syn in a co-culture system consisting of predominant astrocytes and, to a lesser extent, neurons and oligodendrocytes [25]. In contrast to neurons, the astrocytes were found to rapidly ingest large amounts of α-syn. Due to incomplete digestion, intracellular α-syn deposits remained in the astrocytes, which resulted in a mitochondrial impairment [25].

The aim of the present study was to investigate whether α-syn oligomer-selective antibodies can affect astrocytic accumulation of α-syn oligomers in vitro. The oligomer-selective antibodies used in this study have approximately 500-times higher affinity for oligomers compared to monomeric protein [26]. Interestingly, we found that oligomer-selective antibodies effectively prevented astrocytic accumulation of exogenously added α-syn oligomers. Moreover, the antibodies rescued the astrocytes from mitochondrial fragmentation. Our results suggest that immunotherapeutic approaches involving antibodies selective for soluble α-syn oligomers could improve astrocyte functioning, including their neuroprotective effects in alpha-synucleinopathies.

## Methods

### Animals

All animal experiments were approved by the Uppsala County Animal Ethics Board, following the rules and regulations of the Swedish Animal Welfare Agency and were in compliance with the European Communities Council Directive (2010/63/EU). The animals were housed at the Uppsala University Hospital in a 12:12 dark:light cycle. The mice were kept in an enriched environment and given water and food ad libitum. Embryonic C57BL/6 mice were used for the cell culture experiments.

### Neural cell culture

Cortices from 14-day-old mouse embryos (E14) were dissected, and neurospheres were prepared and differentiated as previously described [27]. The cells were expanded in DMEM/F12-GlutaMAX supplemented with 1 × B27, 50 U/ml penicillin, 50 mg/ml streptomycin and 8 mM Hepes buffer; 10 ng/ml bFGF (all from Invitrogen); and 20 mg/ml EGF (VWR). The neurospheres were passaged every second or third day by mechanical dissociation. Prior to experiments, the cells were plated as a monolayer, at a concentration of 1.5 × 10^4^ cells/ml on cell culture dishes or coverslips coated with poly-l-ornithine (0.0025%, Sigma-Aldrich) and laminin (1 μg/ml, Invitrogen). After 24 h, the media was replaced with mitogen-free media (DMEM/F12-GlutaMAX supplemented with 1 × B27, 50 U/ml penicillin, 50 mg/ml streptomycin, and 8 mM Hepes buffer (all from Invitrogen)) to initiate neural stem cell differentiation to a mixed population of neurons, astrocytes, and oligodendrocytes, but not microglia. This is a well-characterized cell culture system, based on the lineage-restricted differentiation of embryonic, cortical stem cells [28–30]. By using immunocytochemistry, we have previously confirmed that the culture (after 1 week of differentiation) consists of a mixed population of neurons (20%), astrocytes (75%), and oligodendrocytes (5%), but importantly no microglia [25]. Neurospheres from passages 1–3 were used for the experiments.

### Generation and labeling of HNE-induced α-syn oligomers

Recombinant α-syn was produced as previously described [31]. Monomeric α-syn (140 μM) was incubated with 4-hydroxy-2-nonenal (HNE) (Cayman Chemicals) in a HNE:α-syn molar ratio of 30:1 at 37 °C for 72 h. To control the ratio of oligomerization, samples were analyzed with SEC-HPLC, previously described in [25]. The characteristics of the oligomers have been thoroughly described [31]. In short, the size of the oligomers is about 2000 kDa and their width is 100–200 nm. Moreover, they are stable and β-sheet rich but do not form fibrils. The α-syn oligomers were labeled with Cy3 using Amersham Cy3 Antibody Labeling Kit (GE Healthcare (cat. # PA33000)). Unbound excess Cy3 was removed by filtration in a Zeba spin desalting column (Thermo Scientific).

### Alpha-synuclein and antibody exposure

Alpha-synuclein oligomers were pre-incubated with the α-syn oligomer-selective antibodies, mAb47 (IgG1) [9], mAb49G (IgG1) [32], or mAb38E2 (IgG2b), generated by the research group [26] at a 1:1 ratio (0.05 μM) in regular growth media at 37 °C for 1 h. The co-cultures were then exposed to 0.05 μM oligomers or to oligomers pre-incubated with antibodies. To analyze the effect of mAb47 on α-syn accumulation further, mAb47 was added after the 24-h α-syn oligomer exposure. The cultures were thoroughly washed in cell culture media prior to the antibody addition and cultured with the antibody for 24 h. In another control experiment, the co-cultures were incubated with mAb47 for 24 h and then extensively washed in cell culture media, prior to 24-h Aβ_42_ protofibril exposure. As negative control antibodies, the irrelevant antibody mAb-Ly128 (IgG_1_, Mabtech), recognizing flagellin in bacteria, were used. For visualization of mitochondria, the cultures were transfected with Cell light Mitochondria-GFP (BacMam 2.0, Life Technologies) during the oligomer exposure. After 24 h of oligomer exposure, conditioned media was collected and the cells were washed in fresh media and fixed or lysed for further analyses. Cells were also recorded using a time-lapse microscope (Nikon Biostation IM Cell Recorder), during exposure to α-syn oligomers (0.05 μM) or to oligomers pre-incubated with mAb47 (0.05 μM, 1:1 ratio). Images (40X) were captured every 10 min for 24 h.

### Lysosomal inhibition

In order to investigate the influence of the endosomal/lysosomal pathway on the antibody-mediated α-syn reduction in astrocytes, cells were pre-incubated with two different lysosomal inhibitors. Co-cultures were incubated with the lysosomal inhibitors bafilomycin (Baf; Calbiochem, Millipore 196000, 0.5 μM) or chloroquine (Chq; Sigma, C6628, 10 μM) for 30 min prior to addition of α-syn oligomers or oligomers pre-incubated with mAb47. The inhibitors remained in the media during the 24-h exposure. Following fixation, the accumulation of α-syn oligomers was assessed by immunofluorescence. The inhibitors did not have any apparent toxic effects in the concentration and exposure times used.

### Immunocytochemistry

Cells were cultured on coverslips and fixed with 4% PFA for 15 min at room temperature (RT). The cells were then permeabilized and blocked for 30 min at RT in 0.1% Triton X-100 + 5% normal goat serum (NGS) in PBS and incubated for 1 h at RT with primary antibodies diluted in 0.1% Triton X-100 + 0.5% NGS in PBS. The primary antibodies used were polyclonal rabbit anti-GFAP (DAKO, 1:400) or monoclonal mouse anti-GFAP (Sigma, 1:400), specific for astrocytes; mAb38F, (1:100), selective for α-syn oligomers [26]; and rabbit polyclonal anti-Lamp-1 (Abcam, 1:200), a lysosomal marker. Following washing 3× in PBS, the samples were incubated with secondary antibodies: goat anti-rabbit IgG (H+L), Alexa Fluor® 488 conjugate antibody; goat anti-mouse IgG (H+L), Alexa Fluor® 488 conjugate antibody; or goat anti-mouse IgG (H+L), Alexa Fluor® 647 conjugate antibody (Life Technologies, 1:200) in 0.1% Triton X-100 + 0.5% NGS in PBS at RT for 1 h. Then, the coverslips were washed and mounted using Vectashield Hard Set with DAPI (Vector Laboratories). Internalization of the α-syn oligomer-selective antibodies was detected by staining of permeabilized samples for mouse IgG with a secondary goat anti-mouse 488 antibody (Alexa, Life Technologies, 1:200).

### ELISA

To assess Ab:α-syn oligomer complex formation, an α-syn oligomer-selective ELISA was used as previously described [26]. A reduced ELISA signal was interpreted as interference due to mAb47 binding to oligomers and obstruction of mAb38F binding, in either the capture or detection steps. In short, oligomers were incubated with or without mAb47 (0.05 μM, 1:1 ratio) 1 h in regular media for 37 °C. High-binding 96-well polystyrene plates (Costar 3590) were coated with the oligomer-selective antibody mAb38F (100 ng/well) diluted in PBS overnight (4 °C). The plate was blocked with 1% bovine serum albumin for 2 h before adding the α-syn oligomer standards (0–250 pM), or cell lysate samples were added to the wells and incubated for 2 h, followed by incubation with biotinylated mAb38F (100 ng/well) for 1 h and Streptavidin-HRP (1:5000, Mabtech) for 45 min. Finally, TMB (Neogen) was used as a substrate and the plates were analyzed at 450 nm (Infinite M1000, Tecan). In addition, a sandwich ELISA was used to quantify the total α-syn levels in the cultures. Conditioned media from three different experiments (*n* = 3) was collected after 24 h of exposure to α-syn oligomers or oligomers pre-incubated with antibody. The ELISA was performed as described above, but using Syn-1 (1 μg/ml, 610,787, BD Biosciences) as capture antibody and FL-140 (1 μg/ml, sc-10717, Santa Cruz Biotechnology) for detection. The samples were diluted 1:200 prior to loading on ELISA wells.

### Immunoprecipitation and Western blot

Conditioned media were collected after 24 h exposure to mAb47+α-syn oligomer and stored at − 70 °C. The media were thawed on ice and incubated with 3-mg magnetic beads (Dynabeads, Novex, Life Technologies) with rotation for 2 h at RT. The beads were washed three times, and the protein was eluted for 10 min at − 70 °C, in the presence of LDS sample buffer and sample reducing agent (Bolt, Life technologies). Gel electrophoresis was carried out with the eluate on a 4–12% Bis-Tris Plus gel (Bolt, Life Technologies) for 28 min at 200 V in SDS MES running buffer (Bolt, Life Technologies). Chameleon kit pre-stained protein ladder (Li-Cor) was used as a standard. Transfer onto a PVDF membrane was performed for 1 h at 20 V (Novex, Life Technologies). The membrane was blocked with Odyssey TBS blocking buffer (Li-Cor) for 1 h at RT. After brief washing in TBS-T, the membrane was incubated with the mouse-anti-α-syn antibody mAb211 (Santa Cruz, sc-12,767, 1:500) in 0.5X Odyssey blocking buffer in TBS-T at 4 °C overnight. Washing (3 × 10 min in TBS-T) was followed by incubation with secondary antibody DyLight 680 goat anti-mouse (Li-Cor, 1:10,000) in 0.5X Odyssey blocking buffer in TBS-T for 1 h (RT). Washing 3 × 10 min in TBS-T was followed by imaging in an Odyssey SA infrared imaging system (Li-Cor). Regular media was used as a negative control, and media containing 0.05 μM α-syn oligomers pre-incubated with mAb47 for 1 h at 37 °C was used as a positive (0 h) control.

### Analyses and statistics

When applicable, the results are presented as mean ± standard deviation. For statistical analyses, two-tailed Mann-Whitney *U* test (GraphPad Prism) was used and the levels of significance were set to *P < 0.05, **P < 0.01, and ***P < 0.001. The total number of observations was used for the statistical analyses. All of the experiments were performed on independent cell cultures, derived from mouse embryos of three different pregnant females. A wide-field microscope (Zeiss AxioImager Z1) was used for quantifications of α-syn accumulation (×40 magnification). The area and intensity of the Cy3-α-syn fluorescence signal was analyzed using ZEN 2012 software. In total, 45 images for each treatment (15 images per sample) were included in the statistical analysis. Values were normalized against the number of viable nuclei. Confocal micrographs were taken with a Zeiss LSM 700 confocal microscope (×63 magnification).

Mitochondrial morphology in cell light mitochondria-GFP transfected cells was quantified manually. The number of cells with disrupted mitochondrial networks was counted and normalized against the total number of transfected, GFP-expressing cells. Cells with mitochondria displaying a low degree of branching, signs of swelling, and a dotted pattern were addressed as cells with a “disrupted mitochondrial network.” In total, 45 images for each treatment (15 images per sample) were included in the analysis that was performed in a blinded fashion. At total of 250 cells or more were counted per sample.

## Results

### Oligomer-selective antibodies prevent accumulation of α-syn in cultured astrocytes

**Fig. 1:**
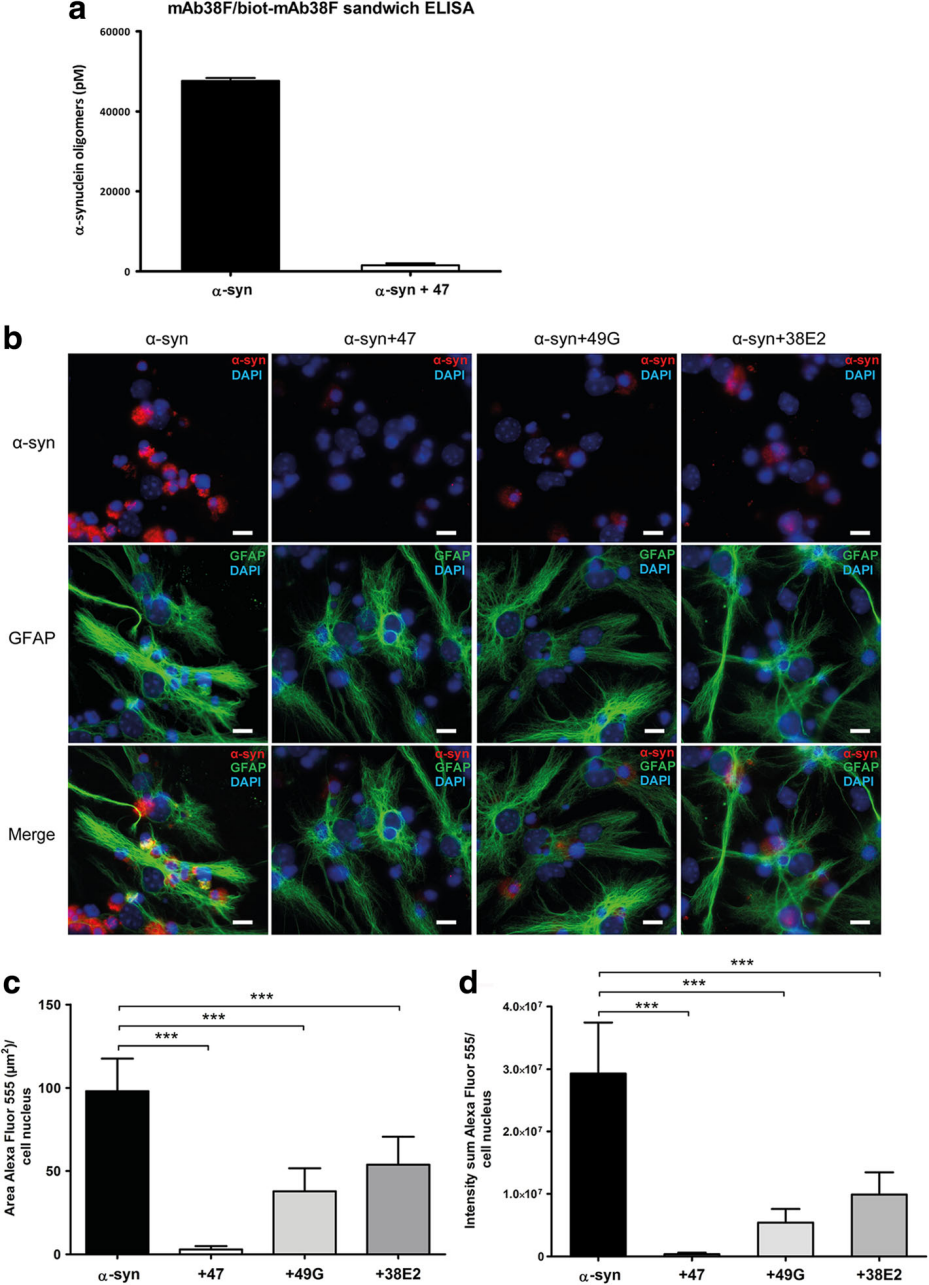
Oligomer-selective antibodies reduce α-syn oligomer accumulation in astrocytes**. a** α-syn oligomers incubated with mAb47 (0.05 μM, 1:1 ratio) 1 h at 37 °C were analyzed with mab38F/biot-mab38F sandwich ELISA. The addition of mAb47 resulted in a strong reduction of the α-syn oligomer signal, implying steric hindrance by mAb47 due to high Ab:α-syn oligomer complex formation. **b** Cells were exposed to fluorescent α-syn oligomers or to oligomers pre-incubated with the α-syn oligomer-selective antibodies: mAb47, mAb49G, or mAb38E2, for 24 h. The α-syn accumulation was strongly reduced when oligomers had been pre-incubated with antibodies. The mAb47 had the most pronounced reducing effect on α-syn oligomer accumulation. Measurements of the intracellular Cy3-labeled α-syn, using the Zen-software, confirmed that the **c** area/cell and **d** intensity/cell were significantly decreased in the antibody-treated cultures. Scale bars = 10 μm. Data are presented as mean ± SD from three independent experiments and levels of significance were set to *P < 0.05, **P < 0.01, and ***P < 0.001

In order to assess the extent of complex formation of antibodies and oligomers prior to cell exposure, an α-syn oligomer-selective sandwich ELISA was used. In the presence of mAb47, there was a 96% decrease in the ELISA signal, demonstrating an almost complete mAb47:oligomer complex formation (hereafter referred to as Ab:oligomer complex) (Fig. 1a).

Co-cultures of astrocytes, neurons, and oligodendrocytes were exposed to Cy3-labeled α-syn oligomers or to Ab:Cy3-oligomer complexes. In the initial analysis, we included three different oligomer-selective antibodies: mAb47 (IgG1) [9], mAb49G (IgG1) [32], and mAb38E2 (IgG2b) [26]. Immunocytochemistry against the astrocytic marker GFAP demonstrated that pre-incubation of oligomers with any of the three antibodies resulted in a reduced α-syn accumulation in the astrocytes (Fig. 1b). Measurements of the intracellular Cy3-labeled α-syn confirmed that the area and the intensity of the α-syn deposits were significantly decreased in the Ab:oligomer complex-treated cultures, as compared to cultures that had been treated with oligomers alone (*p* < 0.0001) (Fig. 1c, d). Since mAb47 displayed the highest capability to reduce astrocytic α-syn accumulation, we focused on this antibody for the subsequent experiments.

### Extracellular antibody-antigen binding is critical for reduced α-syn accumulation

**Fig. 2:**
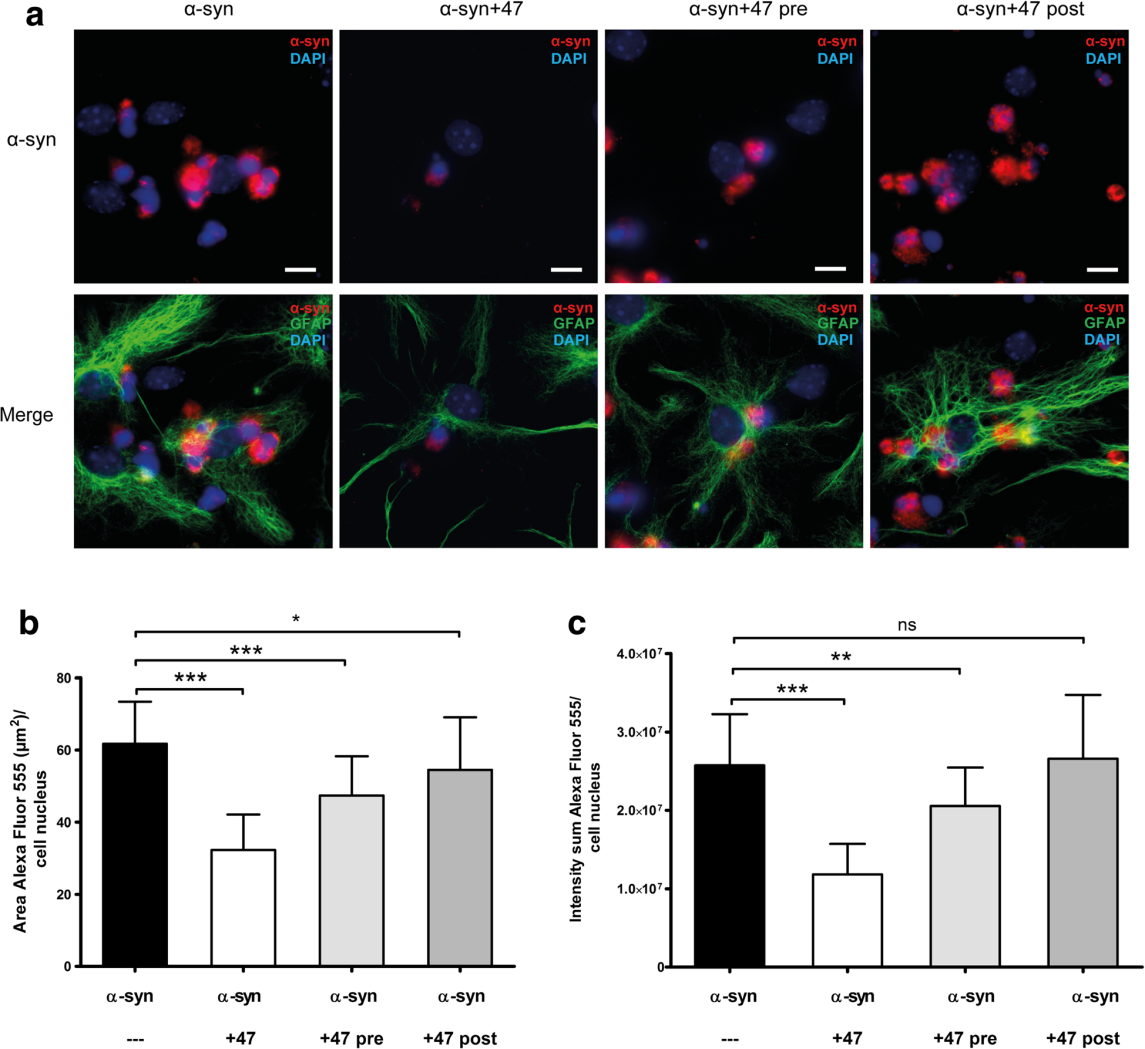
Extracellular antibody binding is critical for the effect on α-syn oligomer accumulation**.** Cells were exposed to Cy3-α-syn oligomers and mAb47 in three different combinations. **a** The cells were either exposed to α-syn oligomers for 24 h (α-syn), resulting in high intracellular accumulation, or to mAb47:oligomer complex (α-syn+47). A clear reduction in α-syn fluorescence signal was detected. Parallel cultures received mAb47 24 h prior to α-syn oligomer exposure (α-syn+47 pre), resulting in a minor reduction of the fluorescence signal. When mAb47 was added 24 h after the oligomer exposure (α-syn+47 post), there was no effect on α-syn accumulation. **b** Fluorescence quantification of Cy3-α-syn area and **c** intensity demonstrated that there was only a significant decrease of α-syn accumulation when mAb47 was added at the same time as the oligomers (α-syn+47) or added before the α-syn exposure (α-syn+47 pre). Scale bars = 10 μm. Data are presented as mean ± SD from three independent experiments and levels of significance were set to *P < 0.05, **P < 0.01, and ***P < 0.001

To investigate if the oligomer-selective antibodies could affect the α-syn accumulation in astrocytes when added to the cells before or after oligomer exposure, cells were exposed to mAb47 for 24 h, either prior to or after α-syn oligomer exposure. For both of these experiments, there were significantly less reduction of astrocytic α-syn accumulation, as compared to the Ab:oligomer complex exposed cultures (Fig. 2a). Quantifications of the area (Fig. 2b) and intensity (Fig. 2c) of the Cy3-α-syn signal demonstrated that the 24 h pre-incubation of the cells with mAb47 resulted in a low, but significant reduction in α-syn accumulation, as compared to exposure to α-syn oligomers alone (area, 20.1% reduction, *p* < 0.0001; intensity, 23.2% reduction, *p* = 0.0029).

**Fig. 3:**
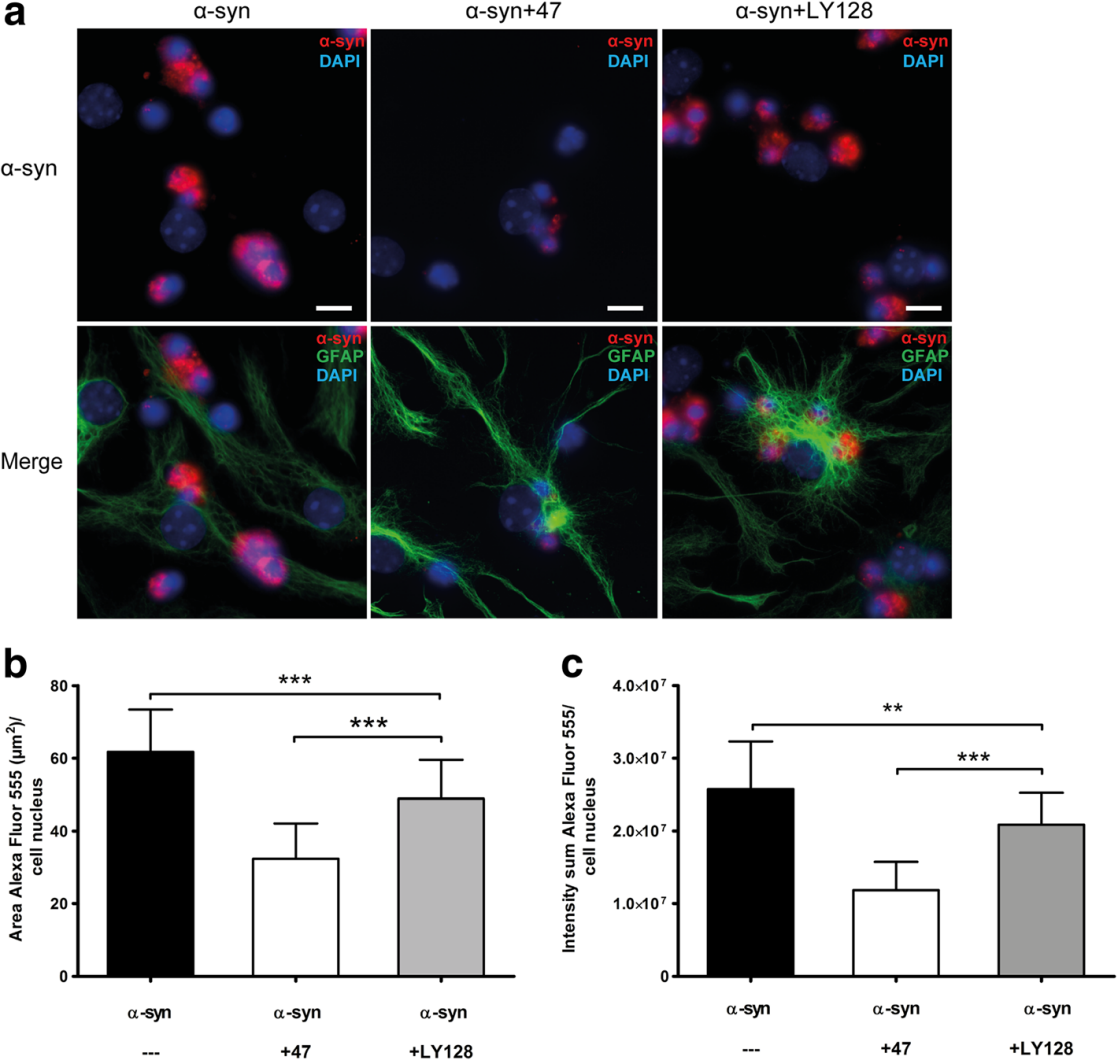
Treatment with irrelevant antibodies has no effect on α-syn accumulation**.** To investigate the specificity of the antibody-mediated reduction of α-syn accumulation, cells were exposed to Cy3-oligomers pre-incubated with an irrelevant antibody of the same IgG subclass as mAb47 (IgG1). The IgG1 LY-128 has no affinity for α-syn and therefore served as an irrelevant isotype control. Alpha-synuclein oligomers were pre-incubated with mAb47 or LY-128 prior to the 24-h cell exposure. **a** The cultures that were treated with mAb47 (α-syn+47) displayed a reduced α-syn accumulation. This effect was significantly stronger than in cultures treated with LY-128 (α-syn+LY128). **b** Quantification of Cy3-α-syn fluorescence area and (**c**) intensity confirmed a significant decrease of α-syn signal in mAb47-treated cultures compared to LY-128-treated cultures. Scale bars = 10 μm. Data are presented as mean ± SD from three independent experiments and levels of significance were set to *P < 0.05, **P < 0.01, and ***P < 0.001

When the mAb47 antibody was added after the oligomer exposure, no reduction in Cy3-α-syn intensity and only a minor reduction in Cy3-α-syn-positive area (11.7% reduction, *p* = 0.0292) could be detected. These results indicate that extracellular antibody-antigen interaction is crucial for an effective antibody-mediated reduction of the α-syn deposits in the astrocytes. To assess the specificity of the observed antibody-mediated effect, we compared mAb47 to an irrelevant IgG1 antibody, LY-128, targeting flagellin in bacteria (Mabtech AB, Nacka Strand, Sweden) [33]. LY-128 was mixed with α-syn oligomers for 1 h at 37 °C and added to the cells for 24 h. Microscopy analyses indicated that the irrelevant IgG had some effect on α-syn oligomer accumulation, but that the mAb47 antibody had a clearly stronger reducing effect (Fig. 3a). Quantification of the area (Fig. 3b) and the intensity (Fig. 3c) of the Cy3-α-syn signal verified that there was a small but significant reduction of α-syn accumulation in the presence of the LY-128 antibody. However, there was a pronounced reduction of α-syn accumulation in the presence of mAb47 (*p* < 0.0001, compared to the effect of LY-128). Our results indicate that the irrelevant antibody can affect the accumulation to some degree, possibly by activating the astrocytes to a more reactive state by binding to their Fc-receptors.

### Intracellular mAb47 co-localizes with α-syn in astrocytes

**Fig. 4:**
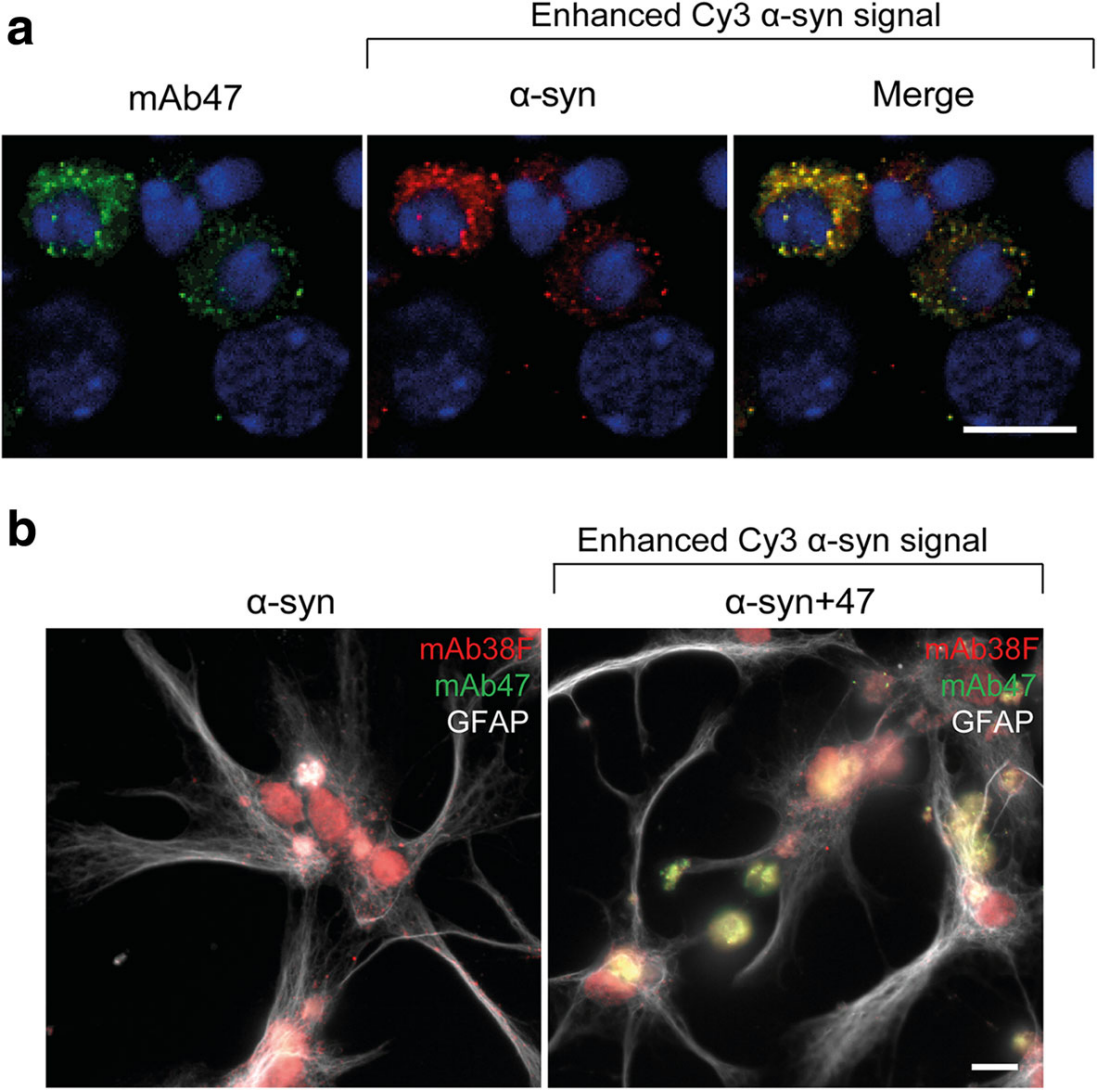
Intracellular mAb47 co-localizes with α-syn oligomers in astrocytes**.** Co-cultures were exposed to Cy3-α-syn oligomers or to Cy3-oligomers pre-incubated with mAb47. **a** Immunofluorescence with secondary antibodies directed towards mAb47 displayed the antibody internalization (green). The Cy3-α-syn signal (red) was weak, indicating low oligomer accumulation. Enhancing the Cy3-α-syn signal revealed intracellular co-localization of oligomers and mAb47 (blue = DAPI). **b** As a control, cells were exposed to non-fluorescent α-syn oligomers that had been pre-incubated with mAb47. Co-staining with antibodies to GFAP (white), mAb47 (green), and oligomeric α-syn (detected by mAb38F, red) revealed co-localization of mAb47 and oligomeric α-syn inside astrocytes. Scale bars = 10 μm

To study the intracellular location of ingested mAb47, the cell cultures were stained with a secondary anti-mouse IgG antibody. Analyses revealed that the ingested mAb47 had perinuclear localization (Fig. 4a). The Cy3-α-syn signal was very weak, but intracellular co-localization of mAb47 and α-syn could be detected when the exposure time of the Cy3-channel was increased (Fig. 4a). The oligomer-selective antibody mAb38F [26] consistently displayed a similar staining pattern and co-localized with the mAb47 signal (Fig. 4b). These results indicate that although most of the Ab:oligomer complexes were cleared, and a small fraction of the Ab:oligomer complexes was still intact and present inside the astrocytes.

### Intracellular α-syn oligomers co-localize with LAMP-1

**Fig. 5:**
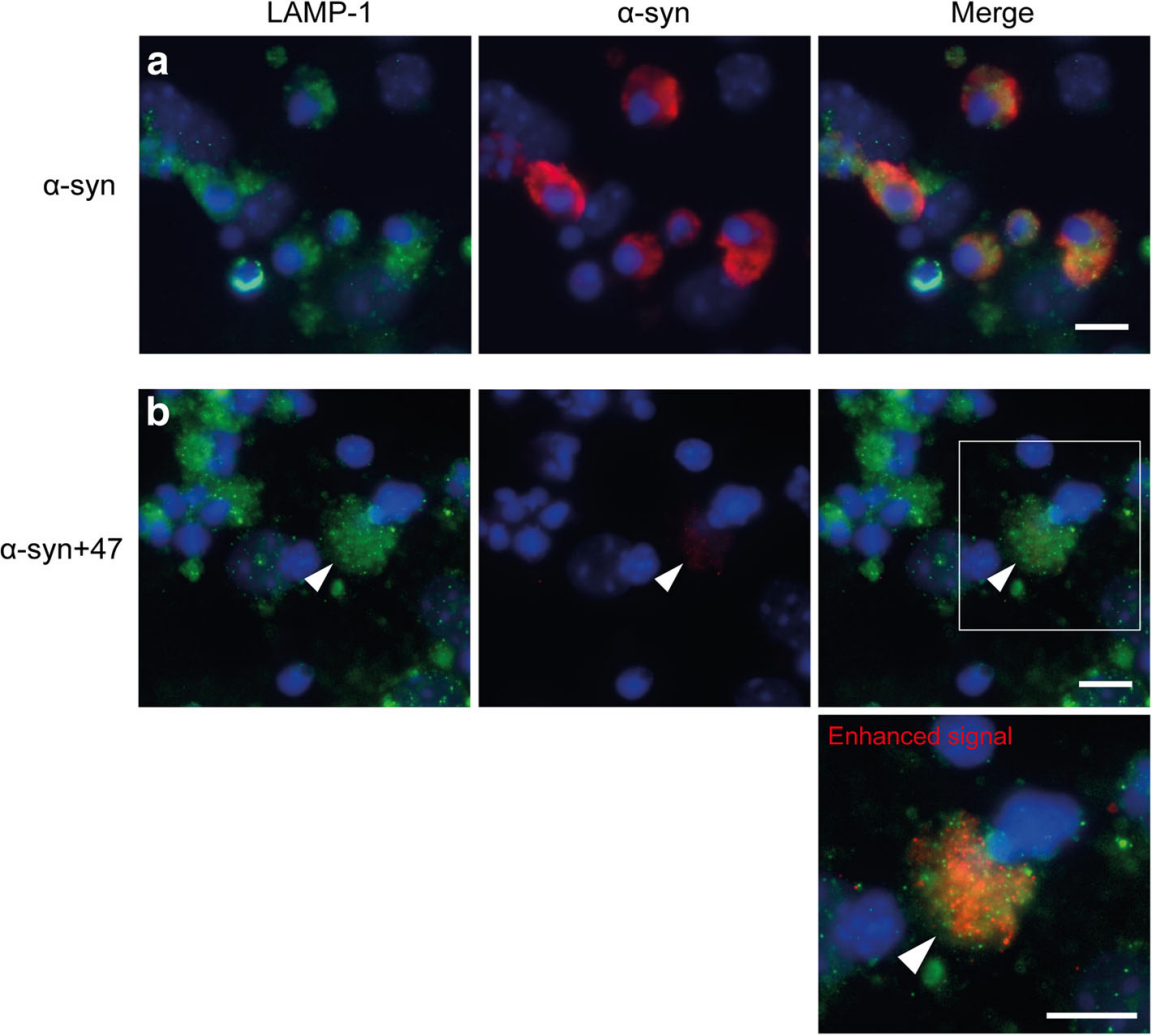
Internalized α-syn oligomers co-localize with lysosomal markers, irrespective of antibody treatment**.** Co-cultures were exposed to either Cy3-α-syn oligomers or Cy3-oligomers pre-incubated with mAb47. **a** After 24 h, oligomers were localized in LAMP-1-positive endosomal-lysosomal compartments. **b** After pre-incubation with mAb47, the levels of intracellular α-syn were very low. However, when the signal was enhanced the Cy3-α-syn still displayed co-localization with LAMP-1 (arrow) (blue = DAPI). Scale bar = 10 μm

In line with previous results (Lindström et al. 2017), α-syn oligomers were found to co-localize with the lysosomal/endosomal marker LAMP-1 after 24 h exposure (Fig. 5a). Also in the Ab:oligomer complex-treated cultures, the ingested α-syn localized to LAMP-1 containing compartments at 24 h (Fig. 5b). However, due to the very low levels of intracellular α-syn in these cultures (in relation to the cultures treated with only oligomers), the signal had to be enhanced to be visible.

### mAb47 rescues astrocytes from mitochondrial network disruption

**Fig. 6:**
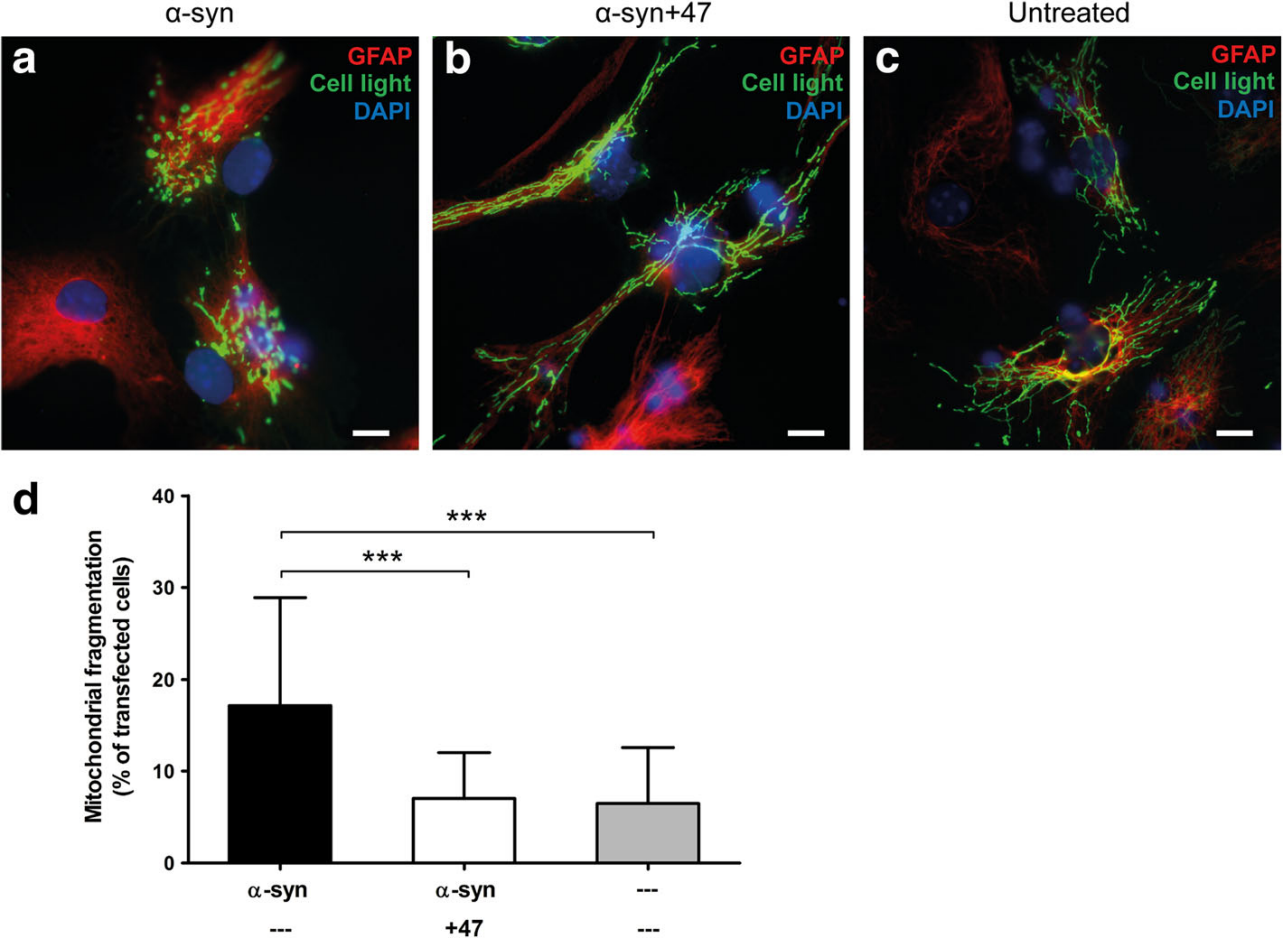
Treatment with mAb47 prevents α-syn oligomer-mediated mitochondrial stress effects**.** During the 24 h exposure to α-syn oligomers or Ab:oligomer complexes, cells were transfected with Cell light Mitochondria-GFP to label the mitochondria. **a** Astrocytes exposed to α-syn oligomers frequently displayed a disrupted mitochondrial network and mitochondrial swelling. **b** The astrocytes in cultures exposed to α-syn oligomers pre-incubated with mAb47 displayed an elongated, branched mitochondrial network throughout the cells, similar to that of **c** non-treated cells. **d** Cells with disrupted mitochondrial networks were counted and normalized against the number of transfected cells. Oligomer exposure led to a clear increase in cells with mitochondrial fragmentation, whereas cultures exposed to oligomers pre-incubated with mAb47 did not significantly differ from the untreated control cells. Scale bars = 10 μm. Data are presented as mean ± SD from three independent experiments and levels of significance were set to *P < 0.05, **P < 0.01, and ***P < 0.001

Labeling of the mitochondria with cell light mitochondria-GFP visualized a distinct mitochondrial swelling and increased mitochondrial fragmentation in astrocytes exposed to 0.05 μM α-syn oligomers (Fig. 6a). In contrast, the mitochondrial networks in astrocytes exposed to the Ab:oligomer complex remained intact (Fig. 6b) as compared to untreated control cells (Fig. 6c). Quantification of fluorescence images revealed a significant increase of cells with fragmented mitochondria after exposure to α-syn oligomers. In the presence of mAb47, the percentage of cells with fragmented mitochondria was similar to control cultures (Fig. 6d).

### Endosomal-lysosomal inhibitors do not alter the antibody-mediated effects

**Fig. 7:**
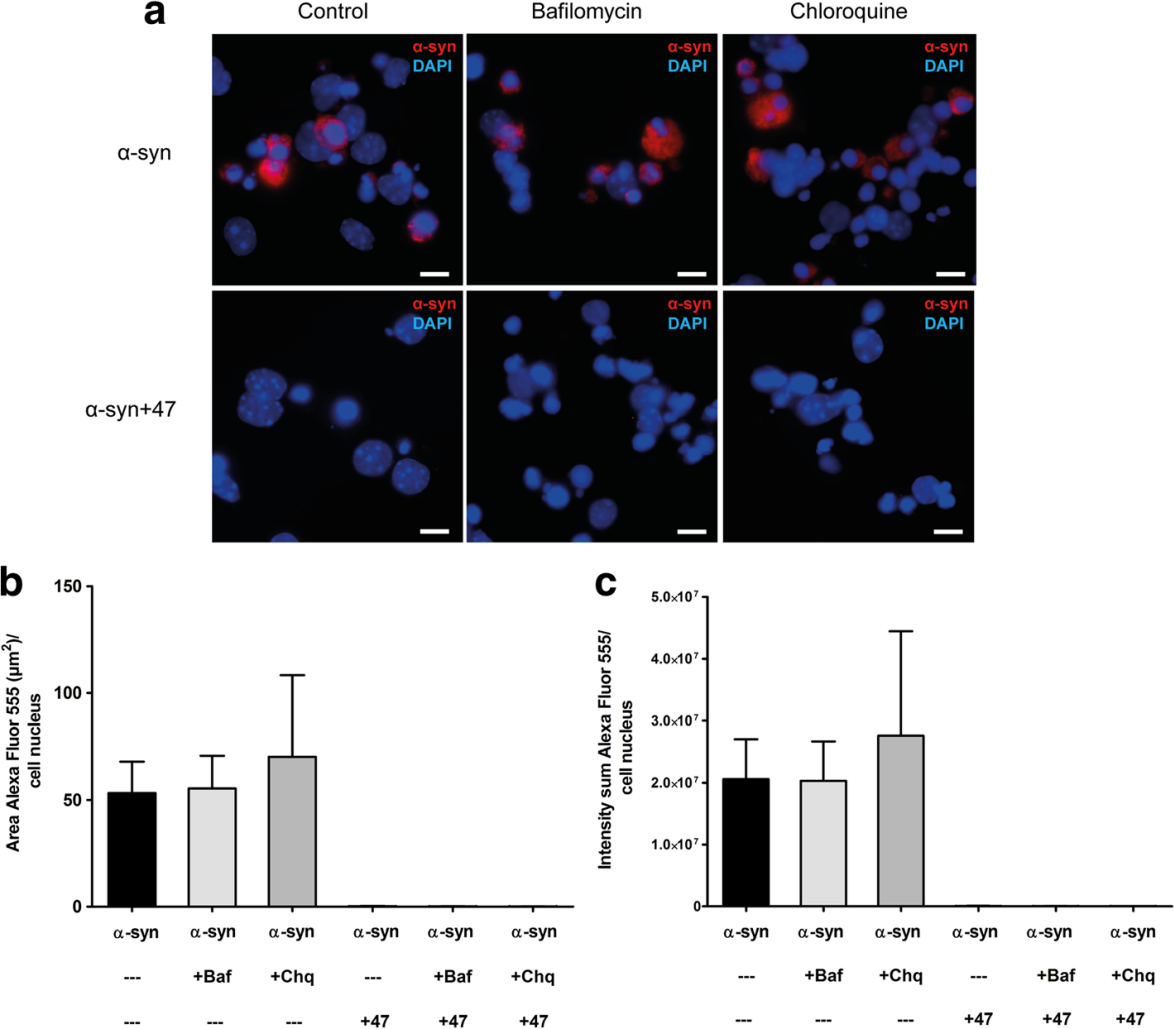
Lysosomal-endosomal inhibition does not alter the antibody-mediated effect on oligomer accumulation**. a** Co-cultures were exposed to either Cy3-α-syn oligomers or to mAb47-treated oligomers for 24 h, in combination with the lysosomal inhibitors bafilomycin (+Baf) or chloroquine (+Chq). The α-syn accumulation was unchanged, both in the absence and presence of mAb47. Hence, chemical inhibition of the lysosomal pathway did not alter the mAb47-mediated reduction of α-syn accumulation. **b** Fluorescence quantification of Cy3-α-syn area and (**c**) intensity confirmed that the inhibitors did not reduce the antibody-mediated effect on α-syn accumulation. Scale bars = 10 μm. Data are presented as mean ± SD from three independent experiments and levels of significance were set to *P < 0.05, **P < 0.01, and ***P < 0.001

In order to investigate the influence of the endosomal/lysosomal pathway on the antibody-mediated α-syn reduction in astrocytes, we pre-incubated cells with the lysosomal inhibitors bafilomycin or chloroquine for 30 min prior to the Ab:oligomer complex exposure. The inhibitors were also present during the 24-h exposure. Neither of the inhibitors affected the mAb47-mediated reduction of α-syn deposits (Fig. 7a). Measurement of the area (Fig. 7b) and the intensity (Fig. 7c) of the Cy3-α-syn signal confirmed that the inhibitors did not interfere with the action of mAb47.

### The presence of mAb47 reduces intracellular α-syn deposits and promotes clearance of extracellular α-syn

**Fig. 8:**
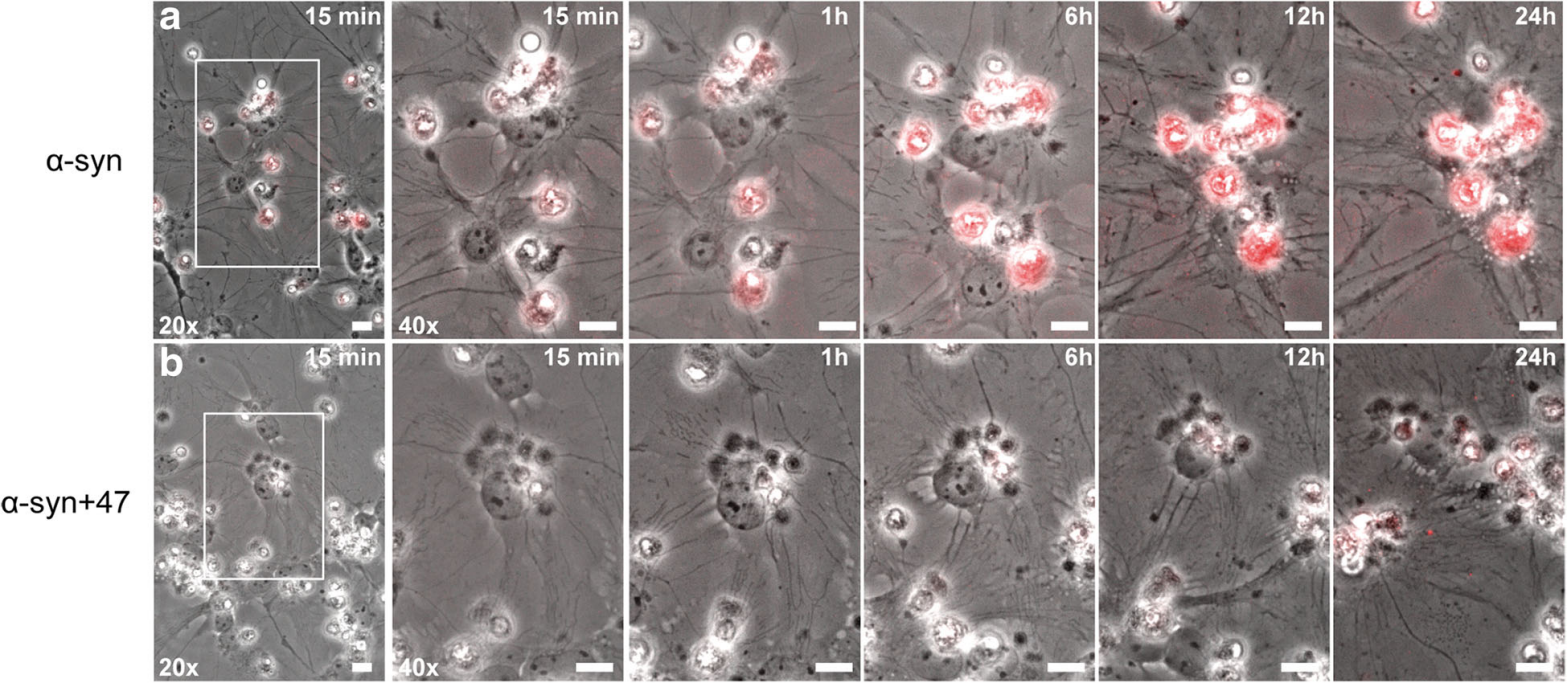
The presence of mAb47 slows down astrocytic accumulation of α-syn oligomers. Co-cultures were exposed to either Cy3-α-syn oligomers or to mAb47-treated oligomers and monitored with time-lapse imaging for 24 h. Areas highlighted with white rectangles to the left (20x) are displayed at higher magnification to the right (40x). When cells were exposed to oligomers alone, the astrocytic accumulation of fluorescent α-syn (red) was rapid, with deposits detectable already after 15 min that were increasing throughout the experiment (**a**). Exposure to mAb47-treated oligomers led to a much lower accumulation in astrocytes. At 24 h, a weak Cy3-α-syn signal was visible in the astrocytes (**b**). Moreover, no free-floating fluorescent aggregates were observed in the cell media, suggesting an overall increased clearance of α-syn oligomers (scale bars = 10 μm)

**Fig. 9:**
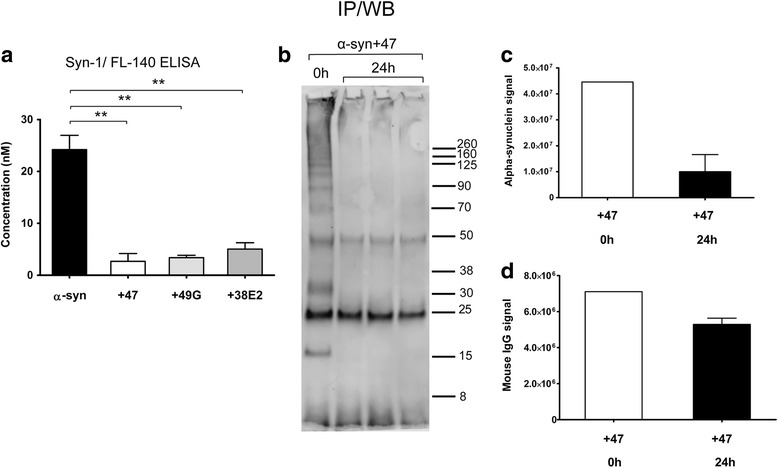
Antibody treatment results in lower extracellular levels of α-syn. Extracellular levels of total α-syn were measured with a sandwich ELISA and immunoprecipitation followed by Western blot. **a** Co-cultures were exposed to α-syn oligomers or to oligomers pre-incubated with mAb47, mAb49G, or 38E2. Conditioned media analyzed with sandwich ELISA demonstrated that the α-syn levels were dramatically decreased when any of the antibodies were present. The largest reduction in extracellular α-syn levels was seen with mAb47. Data are presented as mean ± SD, and the levels of significance were set to *P < 0.05, **P < 0.01, and ***P < 0.001 (*n* = 3). **b** To detect the fraction of α-syn oligomers bound to mAb47, immunoprecipitation was performed on conditioned media from the mAb47-treated sample (24 h). As a positive control, media containing mAb47:oligomer complex that had not been in contact with cells was used (0 h). Western blot on eluted immune complexes displayed a smear of high molecular weight α-syn species, dimer (35 kDa), and monomer bands (14 kDa) in the 0 h control. The 24 h samples showed reduced α-syn signals, as compared to the 0 h control. In addition, the heavy (50 kDa) and light (25 kDa) IgG chains from mAb47 were detected. **c** Quantification of α-syn bands revealed a clear decrease after 24 h, as compared to 0 h. **d** The heavy and light chain bands of mAb47 were quantified and displayed only a modest reduction after 24 h (*n* = 3)

Next, the effect of mAb47 on the total intra- and extracellular levels of α-syn was investigated. For this purpose, time-lapse recordings of co-cultures were performed during the 24-h Cy3-α-syn oligomer exposure. In line with our immunocytochemistry data, the Cy3-α-syn oligomers accumulated inside the astrocytes over time (Fig. 8, upper panel). Already after 15 min, the accumulation of internalized oligomers was evident. However, when cells were exposed to Ab:oligomer complexes, only a very low intracellular accumulation of Cy3-α-syn was observed over time (Fig. 8, lower panel). Moreover, no increase in extracellular Cy3-positive aggregates could be detected in mAb47-treated cultures. In line with that observation, a marked decrease in extracellular α-syn in the presence of the oligomer-selective antibodies mAb47, mAb49G, or mAb38E2 was detected in media analysis using an α-syn sandwich ELISA (Fig. 9a). The concentration of α-syn in media from the mAb47-treated samples had decreased most (86% reduction, *p* = 0.0022), compared to conditioned media from cells exposed to α-syn oligomers only. However, also mAb49G (83% reduction, *p* = 0.0022) and mAb38E2 (76% reduction, *p* = 0.0022) were found to decrease extracellular α-syn.

### Extracellular levels of complex-bound α-syn are reduced whereas mAb47 remains at high levels

To further study the effect of oligomer-selective antibodies on extracellular α-syn clearance, we performed immunoprecipitation on cell culture media. Protein G-coupled beads were used to pull down the Ab:oligomer complexes present in the media. Western blotting of the immune precipitated samples using mAb211 displayed a reduction in extracellular, complex-bound α-syn in the 24 h samples from three cell batches, as compared to the control (Fig. 9b). In the control media (harvested prior to any cell contact), a range of high molecular weight α-syn species were detected as well as monomers (14 kDa) and dimers (35 kDa). In the 24 h samples, the high molecular weight signals had diminished and the monomers or dimers were no longer detectable. Quantification of the total α-syn signal per lane (excluding IgG chain bands) revealed a 77% decrease of the total α-syn signal (Fig. 9c). Interestingly, at both time points, the heavy and light chain bands from mAb47 (at 50 and 25 kDa, respectively) were detected (Fig. 9b). When the signal intensities of the heavy and light IgG chain bands were measured, we found only a minor reduction (26%) in the 24 h samples compared to the 0 h control (Fig. 9d).

## Discussion

Immunotherapy has emerged as a promising method to target α-syn pathology, but the underlying mechanisms and the role of different cell types are poorly understood. Whether astrocytes are involved in the cellular responses to α-syn immunotherapy has not been previously addressed. Here, we demonstrate for the first time that oligomer-selective antibodies can increase clearance of pathological α-syn and maintain healthy mitochondria in astrocytes.

We have previously shown that treatment with the oligomer-selective antibody mAb47 leads to lowered α-syn oligomer levels in the spinal cord of transgenic A30P α-syn mice, correlating with ameliorated motor symptoms [9]. In the present study, we investigated the effects of three different oligomer-selective antibodies: mAb49G, mab38E2, and mAb47, on α-syn-induced pathology in a co-culture system of astrocytes, neurons, and oligodendrocytes. The astrocytes were found to ingest large amounts of oligomeric α-syn that accumulated as intracellular deposits and caused mitochondrial impairment, similar to previous observations [25]. Interestingly, the three oligomer-selective antibodies displayed different capacities in reducing the intracellular α-syn deposits, with mAb47 being the most effective. The varying effects of the antibodies could be due to differences in their interaction with α-syn oligomers in the extracellular milieu or to differences in the interaction of the antibodies to extracellular or intracellular receptors, depending on their different IgG sub-classes [34, 35]. Our pull-down Western blot analysis demonstrated that although the oligomers of the Ab:oligomer complexes were cleared from the culture, the antibodies remained. It could therefore be speculated that the astrocytes actively recycled and secreted the antibodies, whereas the oligomers in the complex were effectively degraded by the cells. Recycling of antibodies has been suggested to be crucial in immunotherapy, by enabling each antibody to bind to its antigen multiple times and thereby increase the clearance of the target protein [36].

In contrast to the α-syn oligomer-selective mAb47, the irrelevant antibody of the same IgG isotype only had a modest effect on the α-syn accumulation. This result indicates that the mechanism of action includes a direct interaction between α-syn and α-syn-oligomer-selective antibodies and is not only the result of a general glial activation (due to antibody binding to the astrocytes’ Fc-receptors). Moreover, addition of the oligomer-selective antibodies before the α-syn oligomer exposure had only a minor effect on the intracellular deposition whereas addition of the antibodies after the oligomer exposure had no effect at all. Taken together, these experiments demonstrated that immune complex formation needs to occur in the extracellular space prior to internalization in order to reduce intracellular α-syn accumulation.

Although the mechanisms responsible for the effectiveness of passive immunization against α-syn pathology are incompletely understood, its beneficial effects have been suggested to include increased clearance of toxic α-syn species from the extracellular space [10], steric hindrance of α-syn aggregation [32], and reduced α-syn propagation between cells [37, 38]. These previous investigations have focused on processes in neurons [38], neuron-like cell lines, or microglia [10], but have not involved astrocytes. Being the most abundant glial cell type in the central nervous system, astrocytes play an important role in maintaining brain homeostasis [20]. In addition, astrocytes respond to neurodegenerative disorders, including PD, through astrogliosis, a process in which they convert to a reactive inflammatory state [23, 39]. The complex role of astrocytes in the pathological brain is largely dependent upon their release and uptake of substances from the micro-environment that they share with the neurons [20]. Reactive astrocytes effectively engulf dead cells, synapses, and protein aggregates of amyloid beta (Aβ) and α-syn [27, 40–46], and we and others have shown that astrocytes may play an important role in spreading of pathogenic proteins [47].

Studies on transgenic mice and cell models have further indicated that anti-α-syn antibody treatment can mediate an upregulation of autophagy [13] and promote lysosomal degradation [10, 48]. In our immunocytochemical analyses, we detected low levels of intracellular α-syn after Ab:oligomer complex exposure. However, by enhancing the fluorescence signal, we could demonstrate that there were still low levels of Ab:oligomer complexes present within Lamp-1-positive structures in the astrocytes, indicating that at least some of the ingested complexes were directed to the endosomal-lysosomal pathway. However, since inhibition of the lysosomal pathway did not have an impact on the reduced accumulation, we speculate that the majority of the Ab:oligomer complexes may be degraded by another route, such as the proteasome pathway or another not yet described mechanism, but additional investigations will be needed to sort that out. Irrespective of degradation pathway, our data indicate that the astrocytic clearance of already ingested Ab:oligomer complexes is rapid. Another possible explanation for the reduced intracellular deposits of α-syn could be a lower uptake or an increased recycling and secretion of the engulfed Ab:oligomer complexes. However, our ELISA and Western blot data of conditioned media showed that both the intra- and extracellular levels of α-syn were lowered in the antibody-treated cultures. In accordance, the time-lapse recordings indicated a treatment-related decrease in the astrocytic accumulation and absence of free-floating Ab:oligomer complexes. Altogether, our data demonstrate that the Ab:oligomer complexes do not accumulate in the astrocytes to the same extent as α-syn oligomers alone, but are rather cleared from the culture.

There have been several reports about how α-syn aggregates induce mitochondrial dysfunction in neurons, including disturbance of the mitochondria fission-fusion homeostasis, autophagosome synthesis, and mitophagy function [49–51]. Our previous results demonstrated a widespread mitochondrial fragmentation and dysfunction in α-syn oligomer treated astrocytes, indicating that the accumulation of α-syn disrupts the autophagosomal and mitophagy machinery [25].

## Conclusions

The present study demonstrates that α-syn oligomer-selective antibodies effectively prevent accumulation of toxic α-syn oligomers in the astrocytes and thereby rescue them from mitochondrial impairment. Neurodegenerative diseases, including PD, are defined by loss of brain homeostasis [22, 52–55], which at least partly could be due to the severely stressed astrocytes that are unable to fulfill their normal tasks [20–22]. Hence, maintaining astrocytic functionality is probably a crucial component for immunotherapy to be successful. In addition, increased astrocytic clearance of soluble α-syn oligomers may reduce PD progression, by preventing further spreading of the α-syn pathology. In order to optimize the design of future antibody-based therapies, we believe that more studies are needed, focusing on various cell types, including astrocytes.

## Abbreviations

Baf: Bafilomycin
Chq: Chloroquine
CNS: Central nervous system
Cy3: Cyanine dye 3
DAPI: 4′,6-Diamidino-2-phenylindole
GFAP: Glial fibrillary acidic protein
GFP: Green fluorescent protein
HNE: 4-Hydroxy-2-nonenal
HPLC: High-performance liquid chromatography
IgG: Immunoglobulin G
IP: Immunoprecipitation
LAMP-1: Lysosome-associated membrane protein-1
LB: Lewy body
mAb: Monoclonal antibody
NGS: Normal goat serum
PBS: Phosphate-buffered saline
PD: Parkinson’s disease
PFA: Paraformaldehyde
PVDF: Polyvinylidene difluoride
SEC: Size exclusion chromatography
TBS: Tris-buffered saline
α-syn: α-Synuclein
